# Effects of Glutamine on Gastric Emptying of Low- and High-Nutrient Drinks in Healthy Young Subjects—Impact on Glycaemia

**DOI:** 10.3390/nu10060739

**Published:** 2018-06-07

**Authors:** Yang T. Du, Diana Piscitelli, Saima Ahmad, Laurence G. Trahair, Jerry R. Greenfield, Dorit Samocha-Bonet, Christopher K. Rayner, Michael Horowitz, Karen L. Jones

**Affiliations:** 1 Endocrine and Metabolic Unit, Royal Adelaide Hospital, Adelaide, SA 5000, Australia; yang.timothy.du@gmail.com (Y.T.D.); michael.horowitz@adelaide.edu.au (M.H.); 2 Adelaide Medical School, The University of Adelaide, Adelaide, SA 5000, Australia; diana.piscitelli@adelaide.edu.au (D.P.); laurence.trahair@adelaide.edu.au (L.G.T.); chris.rayner@adelaide.edu.au (C.K.R.); 3 NHMRC Centre of Research Excellence in Translating Nutritional Science to Good Health, The University of Adelaide, Adelaide, SA 5000, Australia; 4 School of Health Sciences, University of South Australia, Adelaide, SA 5000, Australia; saima.ahmad010@gmail.com; 5 Diabetes and Metabolism Division, Garvan Institute of Medical Research, Sydney, NSW 2010, Australia; j.greenfield@garvan.org.au (J.R.G.); d.samochabonet@garvan.org.au (D.S.-B.); 6 Department of Endocrinology and Diabetes, St Vincent’s Hospital, Sydney, NSW 2010, Australia; 7 St Vincent’s Clinical School, Faculty of Medicine, University of New South Wales, Sydney, NSW 2010, Australia; 8 Department of Gastroenterology and Hepatology, Royal Adelaide Hospital, Adelaide, SA 5000, Australia

**Keywords:** glutamine, gastric emptying, glucose, postprandial, insulin, glycaemia

## Abstract

Glutamine is a potent stimulus for the release of glucagon-like peptide-1, which increases postprandial insulin and slows gastric emptying (GE). We determined the effects of glutamine on GE of, and glycaemic responses to, low- and high-nutrient drinks in eight healthy males (mean age 21.6 ± 0.7 years and BMI 22.9 ± 0.7 kg/m^2^). Participants were studied on four occasions on which they consumed either a low-nutrient (beef soup; 18 kcal) or high-nutrient (75 g dextrose; 255 kcal) drink, each with or without 30 g of glutamine (120 kcal), in a randomised, crossover design. GE (2D ultrasound), blood glucose and plasma insulin concentrations were measured concurrently. Glutamine slowed GE (half emptying time (T50)) of both low- (45 ± 3 min vs. 26 ± 2 min, *p* < 0.001), and high-nutrient, (100 ± 5 min vs. 77 ± 5 min, *p* = 0.03) drinks, however, there was no effect on GE of the high nutrient drinks when expressed as kcal/min (3.39 ± 0.21 kcal/min vs. 3.81 ± 0.20 kcal/min, *p* = 0.25). There was no change in blood glucose after the low-nutrient drinks with or without glutamine, despite a slight increase in plasma insulin with glutamine (*p* = 0.007). The rise in blood glucose following the high-nutrient drink (*p* = 0.0001) was attenuated during the first 60 min by glutamine (*p* = 0.007). We conclude that in healthy subjects, glutamine slows GE of both low- and high-nutrient drinks comparably and attenuates the rise in blood glucose after the high-nutrient glucose drink.

## 1. Introduction

Postprandial hyperglycaemia is a major determinant of overall glycaemic control as assessed by glycated haemoglobin in type 2 diabetes (T2DM). Moreover, its importance increases as glycaemic control normalises [1], such that it is not feasible to achieve “target” glycated haemoglobin levels of ≤7.0% without minimising postprandial glycaemic excursions [2]. Accordingly, strategies to control postprandial hyperglycaemia are pivotal to the management of diabetes, to minimise the risk of the development and progression of microvascular complications. Postprandial glucose may also be a determinant of cardiovascular mortality [3].

It is appreciated that the rate of gastric emptying, which exhibits a substantial inter-, but relatively low intra-, individual variation in healthy subjects and people with diabetes [4], is an important determinant of postprandial hyperglycaemia, accounting for 30–40% of the variance of the initial rise in blood glucose in both healthy subjects [5] and T2DM [6]. This recognition has stimulated the development of dietary [7,8] and pharmacological [9] strategies to improve glycaemic control in T2DM by slowing gastric emptying–the latter including “short-acting” glucagon-like peptide-1 (GLP-1) agonists [10] and the amylin agonist, pramlintide [11]. 

Glutamine, one of the most abundant free amino acids in the human body [12], is widely available as a nutritional supplement. It has been reported that plasma glutamine concentrations are reduced in subjects with T2DM of short duration, who have good glycaemic control [13]. We have reported, in well-controlled T2DM, that acute administration of glutamine in a dose of 30 g decreased the early postprandial glucose excursion after a low-fat meal [14] and administration in a dose of 15 g twice daily for 4 weeks decreased glycaemia, as attested to by a modest reduction in glycated haemoglobin (mean 0.1%) [15]. After oral doses of ~15–30 g, peak plasma glutamine concentrations are achieved at 30–60 min in both healthy subjects and people with T2DM [16]. Glutamine has been reported to be palatable [17] and safe [12] as well as a potent stimulant of the secretion of the incretin hormone, GLP-1 [14,16]. Glutamine is a more potent GLP-1 secretagogue than a number of other amino acids and glucose [18]. GLP-1, released from intestinal L-cells, has a major insulinotropic property, but, because the latter is glucose-dependent, it is not associated with an increased risk of hypoglycaemia [19,20]. Endogenous GLP-1 also slows gastric emptying [21]. Direct stimulation of L-cells by glutamine may also lead to the stimulation of other intestinal hormones, including peptide YY and oxyntomodulin, which are involved in the suppression of appetite and food intake [22,23] as well as GLP-2, which is probably important in the repair of intestinal epithelium [24]. Accordingly, glutamine has inherent, potentially beneficial, metabolic benefits over pharmacological therapies for diabetes such as GLP-1 receptor agonists. 

We have reported that oral glutamine in a dose of 30 g increases GLP-1, glucose-dependent insulinotropic polypeptide (GIP) and insulin levels in lean, obese and T2DM patients [16], however, this study was performed in the absence of a nutrient load. When glutamine (30 g) was consumed with a low-fat meal (5% fat, 230 kcal) in patients with T2DM, postprandial GLP-1 levels were augmented, glycaemia attenuated and insulin levels increased [14]. However, gastric emptying was not measured in these studies. There is limited information about the effect of glutamine on gastric emptying and how this may relate to the associated reduction in glycaemia [25,26]. Lobo et al. reported that gastric emptying, measured by magnetic resonance imaging (MRI), was slowed when 15 g of glutamine was added to a 50 g carbohydrate drink when compared to a 50 g carbohydrate drink without glutamine, but blood glucose levels were not measured [25]. In contrast, Awad et al. [26] reported that 15 g of glutamine in a drink containing 36 g of carbohydrate did not slow gastric emptying (measured by scintigraphy) compared to an isocaloric drink containing 50 g carbohydrate but diminished the glycaemic response. We have reported in both healthy subjects and people with T2DM that intraduodenal infusion of 15 g of glutamine administered immediately before a 75 g glucose drink stimulated phasic pyloric pressures, which is a major mechanism by which nutrients slow gastric emptying [27]. It is not known whether glutamine has different effects on gastric emptying and glycaemia when included with a low-nutrient, compared to a high-nutrient drink/meal, or whether its effects on gastric emptying (and potentially glycaemia) are simply attributable to its caloric content. The rate of gastric emptying is known to be critically dependent on the nutrient content of a meal–low-nutrient liquids empty rapidly in an overall mono-exponential pattern, while emptying of high-nutrient liquids is much slower and approximates a linear pattern [28]. These different patterns reflect the capacity of nutrients to trigger inhibitory feedback arising from the small intestine [29], such that in a given individual the rate of emptying of different drinks/meals when expressed as kcal/min is relatively constant [30]. 

The aims of this study were to determine the effects of 30 g glutamine on gastric emptying of, and the glycaemic response to, low- and high-nutrient drinks in healthy young participants. We employed a non-invasive ultrasound technique which has been validated against the “gold standard” scintigraphy for measurement of gastric emptying [31]. We hypothesised that the addition of glutamine would slow gastric emptying of both low- and high-nutrient drinks and stimulate insulin secretion and, thereby, attenuate the rise in glycaemia in response to the high-nutrient drink.

## 2. Materials and Methods

### 2.1. Study Design and Ethics

This randomized, double-blind, crossover study was conducted in accordance with the Declaration of Helsinki, and the protocol was approved by the University of South Australia Human Research Ethics Committee. All subjects gave their informed consent for inclusion before they participated in the study. Nine healthy males aged between 18–55 years with a BMI of 19–25 kg/m^2^ without a history of abdominal surgery or gastrointestinal disorder were recruited. Subjects were excluded if they had a history of diabetes, smoked (>10 cigarettes/day) or consumed alcohol in excess (>2 standard drinks/day). Habitual exercise habits were evaluated, and subjects asked to maintain these during the study. No subject had regular vigorous exercise.

### 2.2. Interventions

Participants were studied on four separate, randomised, days; on two occasions they received a low-nutrient drink beef soup (18 kcal) comprising Continental^®^ beef stock cubes (Unilever Australasia Ltd., Epping, NSW, Australia) made up to 300 mL with water [32], with or without, 30 g glutamine (120 kcal; l-Glutamine, Cambridge Commodities Ltd. Pty, Cambridgeshire, UK) [16] and on the other two occasions a high-nutrient drink comprising 75 g glucose (255 kcal; Fluka, Sigma-Aldrich^®^ Pty Ltd., Castle Hill, NSW, Australia) made up to 300 mL with water, with or without, 30 g glutamine (120 kcal). Information about the test drinks is provided in Table 1. Each participant attended the laboratory between 8.00–10.00 am after an overnight fast from solids for 14 h and liquids for 12 h. Following placement of an intravenous cannula in an antecubital vein for blood sampling [16], participants were seated in an upright position and instructed to consume the test drink. The drinks were provided at room temperature and consumed within 4 min, with time *t* = 0 defined as the end of drink ingestion. Blood was sampled immediately before ingestion of the drink and at *t* = 15, 30 and 60 min after the low-nutrient, and at *t* = 15, 30, 60, 90, 120 and 180 min after the high-nutrient, drink for measurements of blood glucose and plasma insulin [16]. Serial 2D ultrasound measurements of antral area, to assess gastric emptying, were performed immediately prior to drink ingestion and at 10 min intervals until 60 min after the low-nutrient, and up to 180 min after the high-nutrient drink [32]. Studies were separated by at least 3 days.

### 2.3. Randomisation and Blinding

The randomisation code was generated using an online randomisation program (random.org) [33]. Allocations were then concealed in sequentially numbered sealed opaque envelopes in quadruplicate, which were opened before each arm of the study by a co-investigator who had no involvement in either data collection or analysis. The same co-investigator reconstituted the drinks, transferred them to identical drink vessels wrapped in aluminium foil and gave them to the investigators. It was not possible to fully blind the participants due to the different taste and texture of the low- and high-nutrient drinks. The randomisation code was broken once all data collection and analyses were completed.

### 2.4. Measurement of Gastric Emptying

Gastric emptying was measured using 2D ultrasound by quantifying changes in the stomach antral area using the Antares Sonography System (Siemens Medical Solutions, Mountain View, CA, USA). The accuracy of this technique has been validated by our group against the “gold standard” scintigraphy (r value ~0.95) [31]. Participants were scanned while seated upright at approximately 120 degrees using a C5-2 curved array 2–5 MHz transducer (Siemens Medical Solutions, Mountain View, USA). This body position was maintained throughout the study to exclude the potential confounding effects of posture on gastric emptying and antral area [34]. The transducer was positioned vertically on the skin surface at the level of the xiphisternum and moved inferiorly towards the umbilicus, to visualise the antrum in cross-section with the superior mesenteric vein (SMV) and abdominal aorta as internal landmarks [31]. Data were stored on CD for subsequent measurement by an independent observer with substantial experience in 2D ultrasound measurement of gastric emptying (LGT).

The antral area (cm^2^) was measured using manually operated onscreen callipers. The circumference of the antrum was outlined, and the area recorded during the fasting state (*t* = −2 min) was subtracted from subsequent measurements after drink ingestion. At any time-point, gastric emptying was expressed as:

(1) $$\mathrm{Retention}\;\left(\%\right)=\frac{AA\left(t\right)-AA\left(f\right)}{AA\left(\max\right)-AA\left(f\right)}\times 100$$

where *AA*(*t*) = antral area measured at any given time point, *AA*(*f*) = fasting antral area and *AA*(max) = maximum antral area recorded after drink ingestion [31]. In each study, the 50% gastric emptying time (T50) was determined from a curve of percentage retention vs. time [31], and the rate of caloric emptying, expressed as kcal/min, was calculated based on the T50.

### 2.5. Measurements of Blood Glucose and Plasma Insulin

Blood glucose (mmol/L) was measured using a portable blood glucose meter (MediSense PrecisionTM Q.I.D. System, Abbotts Laboratories, MediSense Inc., Bedford, NY, USA) [16] and plasma insulin (mU/L) by ELISA immunoassay (10-1113, Mercodia, Uppsala, Sweden). The sensitivity of this assay was 1.0 mU/L and the coefficient of variation was 2.9% within, and 5.6% between, assays [35]. 

### 2.6. Statistical Analysis

Statistical analysis was performed with SPSS 16 for Windows (SPSS, Inc., Chicago, IL, USA). Data were analysed using repeated measures two-way Analysis of Variance (ANOVA) for outcomes measured for “treatment” and “time”, with a significance level of *p* < 0.05. If the ANOVA demonstrated a significant “treatment × time” interaction, post-hoc tests were used to examine point-by-point comparisons between treatments, with Bonferroni correction for multiple comparisons. One-way ANOVA was used to analyse the effects of “time” on blood glucose and plasma insulin levels. For the high-nutrient drinks, areas under the curve (AUC) for blood glucose and plasma insulin were calculated using the trapezoidal rule between 0–60 min (AUC0-60) given that the former has been shown to be related to gastric emptying during this time [5,6]. The insulin secretory response was estimated as the ratio of change in insulin (I) to that of glucose (G) at 30 min, represented as ΔI0-30/ΔG0-30 [36]. All outcomes were analysed using Student’s paired *t*-tests. The 95% confidence interval (95% CI) limits for the AUC0-60, gastric emptying T50, blood glucose and plasma insulin concentrations were also determined. Data are presented as mean values ± the standard error of the mean (SEM).

The primary end point of the study was the time to half gastric emptying (T50) of the four test drinks. Secondary end points included the caloric gastric emptying rate, ANOVA of blood glucose and plasma insulin concentrations, as well as AUC0-60 of blood glucose and insulin for the high-nutrient drinks. Assuming an α error of 0.02, to take into the account for potential adjustment for multiple comparisons, power of 82%, effect size of a difference of 2 mmol/L in blood glucose between study days with and without glutamine and a standard deviation of 1.4 mmol/L [16], the required sample size was calculated to be eight. To be conservative we recruited 9 subjects. 

## 3. Results

Of the nine participants, one withdrew due to symptoms of vasovagal pre-syncope following intravenous cannula insertion on the first study day. The remaining eight participants, mean age: 21.6 ± 0.7 years and BMI 22.9 ± 0.7 kg/m^2^, completed the study with no reported adverse events. In one of these, gastric emptying measurements following the high-nutrient drinks were unreliable due to bowel gas artefact and were, accordingly, not included in the analyses.

### 3.1. Gastric Emptying

**Figure 1:**
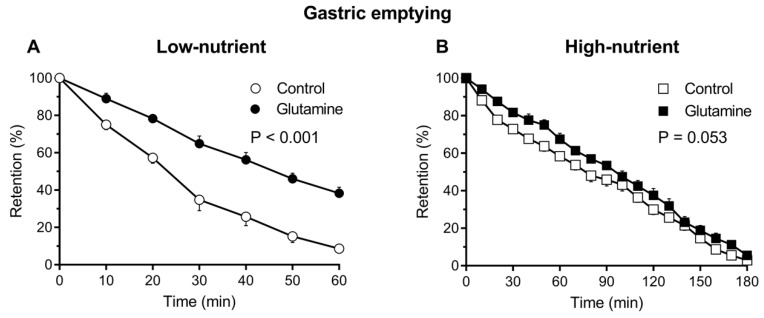
Gastric emptying of (**A**) low-nutrient (○), low-nutrient with glutamine (●) and (**B**) high-nutrient (□) and high-nutrient with glutamine (■). Data are mean ± SEM represented by vertical bars. Ingestion of low-nutrient drink with glutamine slowed gastric emptying significantly compared to low-nutrient drink alone (ANOVA, *p* < 0.001). There was a trend for slowing of gastric emptying of the high-nutrient drink by glutamine (ANOVA, *p* = 0.053).

**Figure 2:**
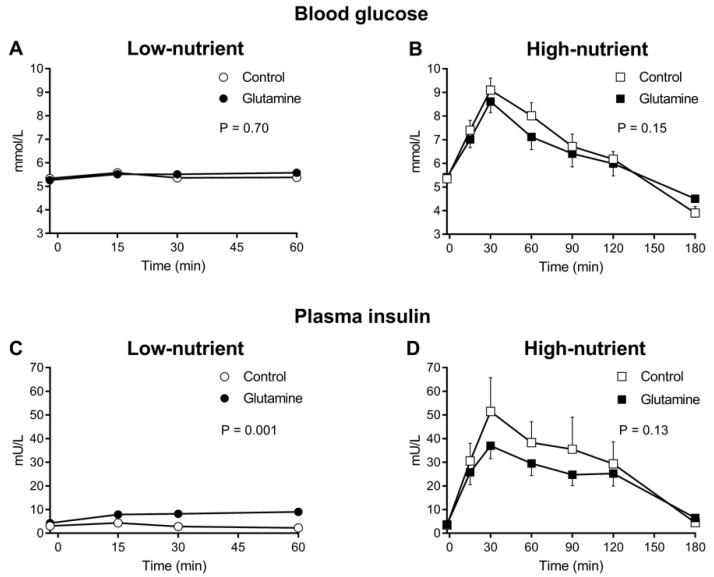
Effects of low-nutrient (○) and low-nutrient with glutamine (●) drinks (ANOVA, *p* = 0.70; (**A**) and high-nutrient (□) and high-nutrient with glutamine (■) drinks (ANOVA, *p* = 0.15; (**B**) on blood glucose. Effects of low-nutrient (○) and low-nutrient with glutamine (●) drinks (ANOVA, *p* = 0.001; (**C**) and high-nutrient (□) and high-nutrient with glutamine (■) drinks (ANOVA, *p* = 0.13; (**D**) on plasma insulin. Data are mean values ± SEM represented by vertical bars.

Gastric emptying of the low-nutrient drinks approximated an overall monoexponential pattern, whereas emptying of the high-nutrient drinks followed a linear pattern. Emptying of the high-nutrient was slower (T50: 77–100 min) when compared to low-nutrient (T50: 26–45 min), drinks (*p* < 0.001 for both). Gastric emptying of the low-nutrient drink was slower (ANOVA: *p* < 0.001) with glutamine (Figure 1A) with an increase in the T50 (45 ± 3 min vs. 26 ± 2 min; *p* < 0.001). The caloric emptying rate of the low-nutrient drink was greater with glutamine (1.53 ± 0.1 kcal/min vs. 0.35 ± 0.03 kcal/min, *p* < 0.001). There was a trend for gastric emptying of the high-nutrient drink (ANOVA: *p* = 0.053) to be slower with glutamine (Figure 1B) with a significant increase in T50 (100 ± 5 min vs. 77 ± 5 min; *p* = 0.026; Figure 2B). The caloric emptying rate for the high-nutrient drinks with and without glutamine, were not different (3.39 ± 0.21 kcal/min vs. 3.81 ± 0.20 kcal/min, *p* = 0.25).

### 3.2. Blood Glucose 

There was no difference in baseline (*t* = −2 min) blood glucose levels on the four days–low-nutrient (without glutamine: 5.3 ± 0.16 mmol/L, with glutamine: 5.3 ± 0.15 mmol/L) and high-nutrient (without glutamine: 5.4 ± 0.15 mmol/L, with glutamine: 5.4 ± 0.17 mmol/L). There was no change from baseline blood glucose (ANOVA) after the low-nutrient drink either with (*p* = 0.15), or without (*p* = 0.24) glutamine and no difference in the blood glucose levels between the two drinks (*p* = 0.70; Figure 2A). 

There was a rise (ANOVA: *p* = 0.0001) in blood glucose after both high-nutrient drinks with and without glutamine and at 180 min, blood glucose was less than baseline following both high-nutrient drinks (*p* < 0.005 for both). The “early” rise (AUC0-60) in blood glucose was less (7.40 ± 0.29 mmol/L.min) with glutamine than without (7.88 ± 0.37 mmol/L.min, *p* = 0.007; Figure 2B). 

### 3.3. Plasma Insulin 

There was no significant difference in baseline (*t* = −2 min) plasma insulin on the four days–low-nutrient (without glutamine: 3.1 ± 0.62 mU/L, with glutamine: 4.3 ± 1.1 mU/L; *p* = 0.27) and high-nutrient drinks (without glutamine: 3.4 ± 0.49 mU/L, with glutamine: 3.7 ± 0.67 mU/L; *p* = 0.56). 

There was no change in insulin after the low-nutrient drink without glutamine, however, insulin levels rose (ANOVA: *p* = 0.007) with glutamine from *t* = 15 min so that plasma insulin was higher after the low-nutrient with glutamine drink compared to without (ANOVA: *p* = 0.001; Figure 2C). Similarly, the AUC0-60 for insulin was greater with glutamine (482 ± 65 mU/L.min) than without (192 ± 32 mU/L.min, *p* = 0.001).

There was a substantial rise in plasma insulin after both the high-nutrient drinks with and without glutamine, which was evident from *t* = 15 min (ANOVA: *p* = 0.001), sustained until *t* = 120 min (*p* = 0.018) and had returned to baseline by 180 min (*p* = 0.095; Figure 2D). There was no difference in the insulin response to the high-nutrient drink with glutamine compared to the high-nutrient drink alone (ANOVA: *p* = 0.13; Figure 2D). Similarly, there was no difference in the AUC0-60 for insulin (1717 ± 233 mU/L.min vs. 2250 ± 549 mU/L.min, *p* = 0.19) or the insulin secretory response (11.1 ± 2.2 mU/mmol vs. 11.6 ± 2.7 mU/mmol, *p* = 0.89) with or without glutamine. 

## 4. Discussion

This study has demonstrated in healthy subjects that glutamine in a dose of 30 g: (a) slows gastric emptying of low- and high-nutrient drinks comparably and (b) attenuates the glycaemic response to a high-nutrient (glucose) drink, attributable to slowing of gastric emptying. These observations support the concept that glutamine may prove useful in the management of T2DM by reducing postprandial glycaemic excursions. 

The observed effect of glutamine to slow gastric emptying of a low-nutrient drink is novel, albeit not surprising. While in some circumstances a large proportion of a low-nutrient drink may empty from the stomach during ingestion [37], in our study the drinks were all ingested within four minutes or less. Slowing of gastric emptying with glutamine was evident from ~10 min and it is, accordingly, likely that this retardation reflects negative feedback triggered by the exposure of the small intestine to glutamine [38,39]. Glutamine also slowed emptying of the high-nutrient drink and the magnitude of this slowing (i.e., change in T50 of ~23 min) was comparable to that observed with the low-nutrient drink (~19 min). Two other studies have evaluated the effect of glutamine on gastric emptying in humans [25,26]. The former reported that gastric emptying (measured by MRI) was slowed (T50 emptying time 78 vs. 47 min, *p* < 0.001) when 15 g glutamine was added to a 50 g carbohydrate drink (234 kcal) when compared to a 50.4 g carbohydrate drink alone (200 kcal). In the latter study there was no effect on gastric emptying (measured with scintigraphy) when 15 g glutamine was added to a 36 g carbohydrate drink (207 kcal) when compared to a 50 g carbohydrate drink alone (207 kcal). In health, the rate of gastric emptying of liquid nutrients is usually in the range of 1–4 kcal/min [40]. The mean prolongation of the T50 of 19 min for the low-nutrient drink and 23 min for the high-nutrient drink by glutamine (120 kcal) is, accordingly, attributable to the caloric content of glutamine rather than any other property. That there was no difference in the rate of emptying of the high-nutrient drinks when expressed as kcal/min is consistent with this concept, which would also explain why Awad et al. [26] failed to find a difference in gastric emptying when glutamine was added, given that the drinks were isocaloric. 

Glutamine has no effect on glycaemia when consumed with the low-nutrient drink despite modest insulin stimulation, consistent with previous findings where blood glucose levels in healthy individuals after ingestion of 30 g glutamine were similar to water [16]. This may potentially reflect the stimulation of glucagon secretion by glutamine, a lack of an insulinotropic effect of GLP-1 because of euglycaemia [14,16] and the potential for glutamine to act as a precursor for gluconeogenesis [16]. The stimulation of insulin is likely to be mediated by plasma glutamine concentrations [17], although, to our knowledge, the effect of parental glutamine on insulin secretion has not been evaluated. l-analyl-l-glutamine, when added to total parenteral nutrition, has been reported to improve glycaemic control and decrease insulin requirement in critically ill patients [41].

The slowing of gastric emptying of the high-nutrient drink by glutamine almost certainly contributed to the reduction in glycaemia. Though we have reported previously an elevation in postprandial GLP-1 levels associated with increased serum insulin levels post ingestion of 30 g glutamine in isolation [16], this was not the case when glutamine was added to a high-nutrient drink. It is known that GLP-1 attenuates the postprandial rise in glycaemia mainly as a result of it slowing gastric emptying, so that glucose medicated insulin secretion is less, resulting in an overall reduction, rather than an increase, in the insulin secretory response to oral glucose [42,43,44]. Our study supports this notion. Other plausible explanations are that the addition of glutamine to a high glucose load increases postprandial insulin-mediated glucose metabolism and disposal without increasing plasma insulin [45,46], and that glutamine affects insulin clearance rather than secretion [14].

We recognise that the observed reduction in glycaemia by glutamine after the high-nutrient drink was modest (and only significant for T50 measurement), but this does not discount the potential for glutamine to be clinically useful in those with T2DM (particularly those with poor glycaemic control) or glucose intolerance given that blood glucose concentrations are much higher in this group. Further studies are indicated. 

Our study has limitations that should be appreciated. We did not determine the effects of glutamine on plasma GLP-1 and glucagon. As all subjects in the study were young healthy males, the observations cannot be generalized to other populations. We studied healthy subjects to avoid the potential confounding effects of disordered gastric emptying, hyperglycaemia and autonomic neuropathy that may occur in people with diabetes. We did not measure C-peptide, which is a better measure of insulin secretion. We only evaluated the effect of one dose of glutamine (30 g) and cannot comment as to whether lower or higher doses may have a similar effect or be more potent (in the case of a higher amount). We have evaluated the effects of glutamine on gastric emptying of single nutrients, and in view of our observations it would be of interest to evaluate the effects of glutamine supplementation on gastric emptying of “normal” mixed solid-liquid meals. Measurement of blood glucose by a glucometer is also less accurate compared with laboratory measurement, especially in the hypoglycaemic range [47]. Lastly, while sonographic measurement of gastric emptying has major advantages given its relative simplicity and non-invasiveness, it is operator dependent [48] and intragastric air has the potential to restrict visualisation of the gastric antrum [32], as occurred in one subject. 

In summary, in healthy subjects, 30 g of glutamine slows gastric emptying of both low- and high-nutrient drinks comparably and reduces the initial glycaemic response to a glucose drink. Glutamine was well tolerated and, while the observed reduction in glycaemia was modest, it has the potential to be substantially greater in subjects with T2DM and such studies are warranted. 

## Figures and Tables

**Table 1:** Components of the four study drinks. The low-nutrient soup drink was derived from Continental ^®^ beef stock cubes (Unilever Australasia Ltd., Epping, NSW, Australia) made up to 300 mL with water [32]. The high-nutrient dextrose drink was derived from 75 g of D-(+)-Glucose monohydrate, Fluka (Sigma-Aldrich^®^ Pty Ltd., Castle Hill, NSW, Australia) made up to 300 mL with water [32].

	Low-Nutrient Soup	Low-Nutrient Soup with Glutamine	High-Nutrient Glucose	High-Nutrient Glucose with Glutamine
Volume (mL)	300	300	300	300
Calories (kCal)	18	138	255	375
Carbohydrate (g)	2.2	2.2	75	75
Fat (g)	0.8	0.8	0	0
Protein (g)	0.4	0.4	0	0
Glutamine (g)	0	30	0	30
